# Ultrafiltration Failure Is a Reflection of Peritoneal Alterations in Patients Treated With Peritoneal Dialysis

**DOI:** 10.3389/fphys.2018.01815

**Published:** 2018-12-20

**Authors:** Raymond T. Krediet

**Affiliations:** Division of Nephrology, Department of Medicine, Amsterdam UMC, Amsterdam, Netherlands

**Keywords:** ultrafiltration failure, fluid transport, vasculopathy, AGE’s, peritoneal dialysis, fibrosis, AQP-1

## Abstract

Ultrafiltration (UF) failure is a common and important complication of peritoneal dialysis (PD), especially in long-term patients without residual urine production, because it often causes overhydration, which is an important cause of death in this population. The current review provides an overview of the pathways of peritoneal fluid transport, followed by the mechanisms and causes of UF failure. The egression of circulating fluid to the tissue compartment and its subsequent re-uptake by the colloid osmotic pressure are markedly influenced by PD, because the dialysis solutions contain glucose as a low molecular weight agent causing removal of fluid from the circulation by crystalloid osmosis. Pores involved in transcapillary UF consist of inter-endothelial small pores and the intra-endothelial water channel aquaporin-1. The former allows transport of plasma fluid with dissolved low molecular weight solutes and accounts for 60% of the filtered volume, the latter transports 40% as pure water. This free water transport (FWT) is driven by the crystalloid pressure gradient, while small pore fluid transport (SPFT) is dependent on both hydrostatic and crystalloid osmotic pressure. The number of perfused peritoneal microvessels as assessed by small solute transport parameters, is differently associated with UF: a positive relationship is present with SPFT, but a negative one with FWT, because the effect of more vessels is counteracted by a faster disappearance rate of glucose. Ultrafiltration failure can be present shortly after the start of PD, for instance due to mesothelial-to-mesenchymal transition. Late UF failure develops in 21% of long-term patients. Both FWT and SPFT can be affected. Patients with encapsulating peritoneal sclerosis have severely impaired FWT, probably due to interference of interstitial collagen-1 with the crystalloid osmotic gradient. This mechanism may also apply to other patients with reduced FWT. Those with mainly impaired SPFT likely have a reduced hydrostatic filtration pressure due to vasculopathy. Deposition of advanced glycosylation end products is probably important in the development of this vasculopathy. It can be concluded that long-term UF failure may affect both SPFT and FWT. Vasculopathy is important in the former, interstitial fibrosis in the latter. Measurements of peritoneal transport function should include separate assessments of small pore-and FWT.

## Introduction

Ultrafiltration (UF) failure during peritoneal dialysis (PD) is the commonly used term for a situation, where netUF, i.e., the difference between the drained and the instilled volume is less than expected in the PD population. As the glucose concentration of the dialysis solution is an important determinant of net ultrafiltration (netUF) by osmosis, normal values are related to the glucose concentration of the dialysate. After a 4 h exchange with 1.36% glucose median netUF was −85 mL (95% confidence interval −454 to +286) in 83 prevalent patients (Pannekeet et al., 1995) and 635 mL (range 95–1305) in 80 prevalent patients after a similar exchange with a 3.86% glucose dialysis solution (Parikova et al., 2005). The extremely large interindividual variability contrasts with an intraindividual variability of about 20% (Imholz et al., 1978) and points to large differences in transport characteristics of the “peritoneal membrane” between individual patients. The International Society for PD published a guideline on UF failure in 2000, containing a definition of UF failure, based on the results of the above mentioned studies: the 3 × 4 rule (Mujais et al., 2000). It states that UF failure is present when netUF is less than 400 mL after drainage of a 4% (3.86 or 4.25%, depending on the pharmacopeia) dialysis solution with an intraperitoneal stay of 4 h. Although patients with UF failure according to this definition are often hypervolemic, overhydration is not included in the definition of UF failure, because overhydration is not only dependent on fluid removal, but also on fluid intake. Yet overhydration is probably the most important cause of cardiovascular death in PD patients (Krediet and Balafa, 2010). As the vast majority of patients with UF failure is hypervolemic, knowledge of the mechanisms for UF failure, its presence, development, diagnosis, treatment and prevention are important.

The aim of the present review is to give an update on pathways and mechanisms of peritoneal fluid transport, and the way functional characteristics can be used in patient’s follow-up to identify the most important cause for UF failure in an individual patient and the mayor underlying morphological abnormalities.

## Pathways for Peritoneal Fluid Transport

According to Starling’s law fluid with dissolved crystalloids egress from circulating blood at the arteriolar side of microcirculatory capillaries to the interstitium of perfused tissues by the hydrostatic pressure, after which the majority is taken up at the venous part by the increased intravascular colloid osmotic pressure and partly into the lymphatic system. Crystalloid fluid administered into the peritoneal cavity increases the intraperitoneal pressure, which is also transmitted to the peritoneal interstitial tissue and will thereby lower the hydrostatic filtration gradient to some extent. The protein content of such solution is so low, that extensive reabsorption by the intracapillary colloid osmotic pressure occurs and also into the lymphatic system. This leads to a complete absorption of all intraperitonealy administered isotonic solutions.

The objective of PD is removal of excess uremic waste products and fluid from the body by drainage of an intraperitoneally administered solution, usually called the dialysate. This is only possible when the reabsorption of filtered fluid is minimal, allowing net transport of fluid and solutes from the tissues to the dialysate, which can subsequently be drained out of the peritoneal cavity. The magnitude of filtration is partly dependent on the systemic blood pressure, and more importantly on autoregulation of organ perfusion by the tonus of the precapillary sphincter in afferent arterioles. Peritoneal diffusion of solutes is another transport mechanism to this hydrostatically induced convection, because the peritoneal cavity is filled with a dialysis solution. The distribution of this electrolyte-containing fluid is not restricted to the peritoneal cavity, but extends into the interstitial tissue up to the vascular wall. This implies that diffusion of solutes from the vascular lumen to the dialysis fluid occurs, which is quantitatively more important than hydrostatic convection, because of the large concentration gradient between blood and dialysate. Addition of a low-molecular weight solute, e.g., glucose to dialysis fluid creates a crystalloid osmotic pressure gradient which is temporary, because the osmotic agent diffuses into the interstitium and from there back into the systemic microcirculation. Together with the hydrostatic pressure gradient, the glucose-induced crystalloid pressure gradient induces fluid transport from the microcirculation to the peritoneal cavity, usually called UF.

According to the 3-pore theory of transport through the microvascular wall (Rippe, 1993), interendothelial pores are the pathways involved in solute transport. These consist mainly of small pores with radii of about 40 Å. The number of large pores (radius > 250 Å) is so small that their contribution to fluid transport and that of low molecular weight solutes can be neglected. Low molecular weight solutes pass the small pores easily, because their molecular radii are about 3 Å and even that of β_2_-microglobulin is only 16 Å. Consequently glucose that has a molecular radius of 3.12 Å, can only induce a limited crystalloid osmotic pressure gradient across the small interendothelial pores. This requires extremely high dialysate concentrations and lasts for a limited length of time, because of its absorption. We found that small pore water transport rates after the first one to two hours of a 3.86% glucose exchange averages 3 mL/min (Parikova et al., 2008). This value remains stable during the remaining 2–3 h (Parikova et al., 2008). The difference between the crystalloid and colloid pressure gradient during the last part of the dwell decreases from 16 to 2 mmHg during the last 2 h of a dwell (Parikova et al., 2008). This is a low value compared to the hydrostatic pressure gradient of 17 mmHg, the latter calculated as the difference between the pressure in peritoneal capillaries which averages 25 mmHg at the arteriolar side (Struijk et al., 1996) and the intraperitoneal pressure of 8 mmHg (Guyton, 1981). Given a net combined crystalloid and colloid osmotic pressure gradient in the initial phase of a 3.86% glucose exchange of about 50 mmHg (Parikova et al., 2008), the contribution of the hydrostatic pressure gradient to small-pore fluid transport will increase from about 34% in the beginning to about 80% during the last 2 h of the dwell.

Besides transcapillary UF by the crystalloid osmotic and hydrostatic pressure gradients which both increase intraperitoneal volume, fluid egresses from the peritoneal cavity by colloid osmosis-induced backfiltration at a rate of 0.4 mL/min (Imholz et al., 1993) and by absorption into the lymphatic system. Assessment of the latter requires intraperitoneal administration of a macromolecule, which is so large that its diffusion can be neglected. Radiolabeled albumin and neutral dextrans have been employed in PD patients (Krediet et al., 1991; Heimburger et al., 1992). The obtained values are dependent on the use of either the disappearance rate of the macromolecule from the peritoneal cavity or its appearance rate in the circulation. Typical values for the disappearance rate are 1.0–1.5 mL/min in PD patients, while they average 0.2 mL/min for the appearance rate (Rippe et al., 1986). The difference is partly explained by underestimation of the appearance rate, because the albumin space is about twice that of the circulating volume, and also by overestimation by the disappearance rate by transmesothelial passage of the macromolecule (Krediet, 2004).

Transcapillary UF by crystalloid osmosis occurs not only through small interendotheliaal pores, but also by the intra-endothelial water channel aquaporin-1 (AQP-1; Stelin and Rippe, 1990; Ni et al., 2006). This transcellular membrane protein functions as an ultrasmall pore, allowing transport of water, but not of solutes, including glucose and Na^+^. Consequently it functions as an ideal semipermeable pore with a refection coefficient of 1.0, while that to glucose of the small pores is only 0.03–0.04 (Imholz et al., 1994). AQP-1 therefore induces free water transport (FWT) by crystalloid osmosis. It explains the so-called sodium sieving, found in an old clinical observation in severely overhydrated patients with acute kidney insufficiency, who were treated with PD using dialysate with very high glucose concentrations (Nolph et al., 1969). These patients showed a decrease of the dialysate Na^+^ concentration, while the plasma concentration remained unaltered. In retrospect this must have been caused by dilution due to high FWT rates.

Assessment of the dialysate/plasma (D/P) ratio of Na^+^ after 60 min of a dialysis exchange has been proposed as a semiquantitative method for AQP-1 function (Mujais et al., 2000). Calculation of FWT_0-60__min_ in patients is also possible using sodium transport in that period: the drained volume is divided in the volume that accompanies Na^+^ transport from the circulation to the dialysate which is assumed to have occurred through the small pores, the remaining part of the drained volume is considered to represent FWT (Smit et al., 2004; La Milia et al., 2005). Transcapillary UF during the initial 60 min can therefore be considered to be composed of small pore fluid transport (SPFT) and FWT. Calculations in patients show that SPFT accounts for 60% of UF and FWT for 40% (Parikova et al., 2005). These values are in close agreement to previous results of computer simulations (Rippe et al., 1991).

## Mechanisms and Causes of UF Failure

**FIGURE 1:**
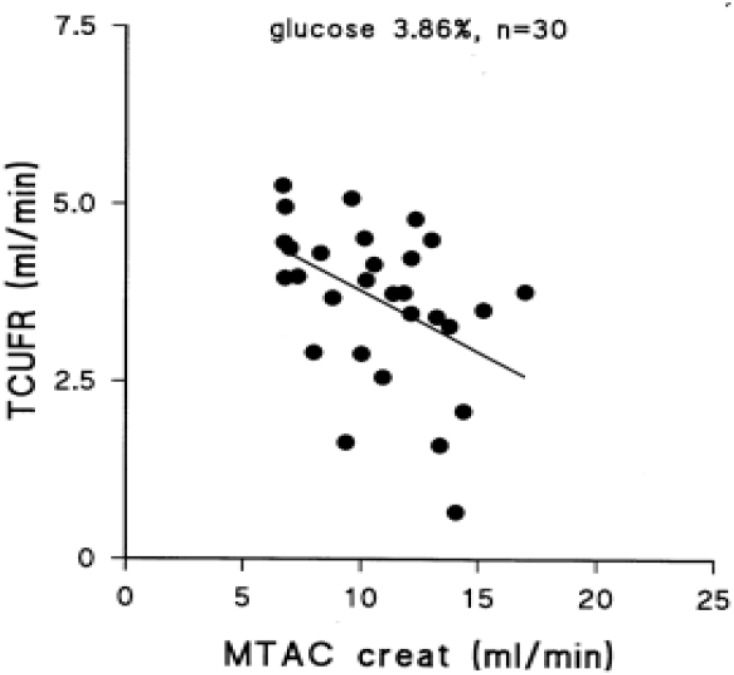
The negative relationship between the transcapillary ultrafiltration rate during a 4 h exchange with 3.86% glucose dialysis solution and the mass transfer area coefficient of creatinine, representing the effective peritoneal vascular surface area. This figure was part of Figure 6.8 of chapter 6 in Krediet (2009). Published with permission of the author and of Springer Science and Business media.

**FIGURE 2:**
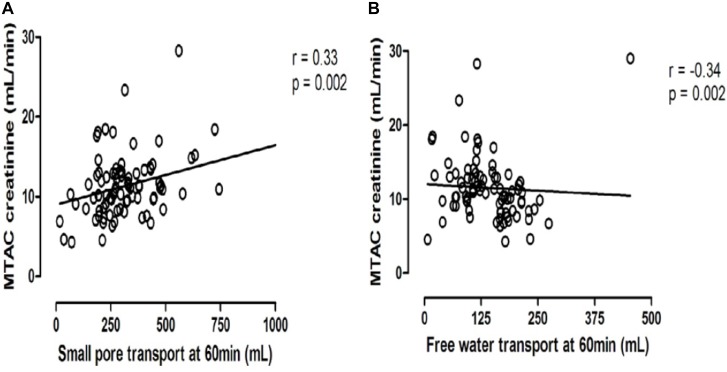
The opposite relationships between the mass transfer area coefficient of creatinine and either small pore fluid transport **(A)** or free water transport **(B)**.

Peritoneal transport of a solute from the circulation to the dialysate-filled peritoneal cavity is dependent on its diffusion velocity mainly determined by molecular weight, and the number of perfused peritoneal microvessels so the number of pores available (Krediet et al., 1993). Comparison of the small pore radius with those of low molecular weight solutes points to the importance of the effective peritoneal surface area (EPSA), i.e., the number of perfused peritoneal capillaries in the determination of the mass transfer area (MTAC) of a solute. This representation of the maximal transport of a solute by diffusion at time zero is often replaced by the D/P ratio after drainage of the dialysate, which is usually performed after a dialysate dwell time of 4 h. Creatinine is usually used as marker solution, because no evidence of local peritoneal production or release is present. Therefore changes in the MTAC creatinine or D/P creatinine represent changes in EPSA. High values of MTAC or D/P creatinine indicate fast peritoneal solute transport including that of glucose, and thereby a rapid disappearance of the crystalloid osmotic gradient and impaired UF. Consequently an inverse relationship between MTAC creatinine and netUF is present, as shown in Figure 1. This is also the case for FWT, but for SPFT a positive relationship is present with MTAC creatinine, as shown in Figure 2. This can be explained by the limited dependence of SPFT on crystalloid osmosis, compared to that on the hydrostatic pressure gradient.

Ultrafiltration failure can be distinguished into early (<2 years) of PD and late UF failure (Sampimon et al., 2011). Early UF failure occurs in about 4% of incident PD patients and is often not a clinical problem, because most patients still have urine production, which protects them for overhydration. With the exception of a high lymphatic absorption rate, which is rarely present, a large EPSA is usually present, not caused by a large number of microvessels, but by increments in their perfusion. EPSA is not a static property of the peritoneal membrane, but fluctuates due to intraperitoneally produced or released vasoactive factors. Acute peritonitis is the best example. It is characterized by a low UF rate and a high MTAC creatinine, caused by peritoneal hyperemia (Krediet et al., 1987; Douma et al., 1998) and mediated by vasoactive substances, like prostaglandins and interleukin-6 (Il-6; Zemel et al., 1993). The higher dialysate than plasma values of these factors point to intraperitoneal production. Peritonitis is an acute and largely reversible condition (Van Diepen et al., 2015), but also microinflammation likely influences peritoneal solute transport, as evidenced by relationships between dialysate Il-6 and D/P creatinine (Pecoits-Filho et al., 2006; Lambie et al., 2013). No association is present with systemic inflammation (Lambie et al., 2013). The increased EPSA, associated with acute peritonitis and with chronic micro inflammation impairs UF, because of the rapid disappearance of the crystalloid osmotic gradient. Epithelial-to-mesenchymal transition of mesothelial cells (MMT) is present in *ex vivo* obtained mesothelial cells from peritoneal effluent of PD patients (Yanez-Mo et al., 2003). Histology studies of peritoneal tissues in PD patients also show this phenomenon. It occurs during the first two years of treatment and is associated with an increased EPSA (Del Peso et al., 2008) and high dialysate concentrations of vascular endothelial growth factor (VEGF; Aroeira et al., 2005). Previously an association between dialysate VEGF and MTAC creatinine has been shown in a cross-sectional analysis (Zweers et al., 1999). A clinical diagnosis of MMT without histologic confirmation is likely when early UFF is associated with high values of MTAC creatinine, effluent VEGF and cancer antigen 125, a marker of mesothelial cell mass or turn-over (Van Esch et al., 2004).

Late UFF develops in about 21% of patients who are treated with PD for >2 years (Sampimon et al., 2011). It involves both FWT and SPFT (Coester et al., 2014). FWT remains stable during the first 3 years of PD, but a subsequent decrease of FWT_0-60__min_ occurs to 67% of the initial value. This is accompanied by an increase of small solute transport that mirrors FWT: MTAC creatinine rises from 10 to 13 mL/min and glucose absorption after 4 h augments from 63 to 69% (Coester et al., 2014).

Very low values for FWT are present in patients with encapsulating peritoneal sclerosis. This is a rare, but severe complication of long-term PD, which occurs in 3% of incident PD patients in the Netherlands after a duration of 5 to 13, mean 8 years (Sampimon et al., 2011), but it occurs more often in Japan (Kawanishi et al., 2004). EPS is clinically characterized by signs of bowel obstruction and morphologically by a thickened peritoneal interstitium with sclerotic changes, leading to adhesion of bowel loops. Especially the deposition of collagen-1 is extremely dense and is associated with osmotic water transport, assessed semiquantitatively, despite a normal expression of AQP-1 (Morelle et al., 2015). Also quantitative values for FWT_0-60__min_ are very low: we found an interquartile range of 24 to 73, median 26 mL (Sampimon et al., 2014). FWT_0-60__min_ < 75 mL predicted EPS with a sensitivity of 100% and a specificity of 81% (Lopes Barreto et al., 2018). Why EPS is associated with low FWT, is still unsolved. AQP-1 function may be impaired, but the deposition of interstitial collagen-1 is probably more important in the function of the crystalloid osmotic gradient, although the mechanism is still unsolved. The use of FWT in the follow-up of PD patients to identify those with progressive interstitial fibrosis has been proposed, but is hampered by the absence of a good reference method for quantification of peritoneal fibrosis (Krediet et al., 2016). The time course of SPFT, the other constituent of transcapillary UF, is different. It shows a gradual decline to 46% of the initial value at 5 years (Coester et al., 2014). The influence of the crystalloid pressure gradient on this decrease can be neglected, compared to that of the hydrostatic pressure gradient, as shown previously. A reduction in the hydrostatic filtration pressure has been hypothesized, due to progressive vasculopathy (Krediet et al., 2018). This condition has first been described in the report of the peritoneal biopsy registry (Williams et al., 2002). Four grades are distinguished, ranging from subendothelial hyalinosis to luminal distortion and even obliteration. It is present in 70% of peritoneal biopsies after PD for more than 5 years. Vasculopathic blood vessels have a narrowed lumen and are therefore likely to lower the filtration pressure and thereby reduce SPFT, in contrast to FWT in EPS which is not related to vasculopathy (Morelle et al., 2015). Similar to diabetic microvascular disease, deposition of advanced glycosylation end products (AGEs) in the vascular wall may be the major culprit for the development of peritoneal vasculopathy. AGEs are irreversible covalently bound complexes between glucose molecules and proteins. *In vivo* they are formed within the vascular wall and lead to protein cross-links in it, thereby increasing its rigidity. AGEs are important in diabetic microangiopathy and impaired kidney function (Makita et al., 1991). AGE deposition is also present in peritoneal tissue of PD patients, especial submesothelial and in the vascular wall (Nakayama et al., 1997; Combet et al., 2000). An association between UF failure, peritoneal AGE deposition and the severity of vasculopathy has been described (Honda et al., 1999).

## Conclusion

The development of UF failure in patients solely treated with conventional PD solution, is a major problem for the long-term use of this mode of renal replacement therapy in end stage renal disease patients without residual urine production. Peritoneal fibrosis and vasculopathy are the most important structural abnormalities, involved in its pathogenesis. FWT is especially dependent on the number of perfused peritoneal blood vessels and probably on the amount of fibrosis, SPFT is reduced by vasculopathy. A reduction of both FWT and SPFT is likely to occur in the majority of long-term PD patients. Those who develop EPS should be considered as a separate subgroup, in which FWT is much more impaired than SPFT. Measurement of peritoneal transport function should not only include netUF, but also separate determinations of FWT and SPFT to guide treatment options.

## Author Contributions

RK wrote this review.

## Conflict of Interest Statement

The author declares that the research was conducted in the absence of any commercial or financial relationships that could be construed as a potential conflict of interest.

## References

[B1] Aroeira L. S. Aquillera A. Selgas R. Ramirez-Huesca M. Perez-Lozano M. L. Cirugeda A. (2005). Mesenchymal conversion of mesothelial cells as a mechanism responsible for high solute transport rate in peritoneal dialysis: role of vascular endothelial growth factor. *Am. J. Kidney Dis.* 46 938–948. 10.1053/j.ajkd.2005.08.011 16253736

[B2] Coester A. M. Smit W. Struijk D. G. Parikova A. Krediet R. T. (2014). Longitudinal analysis of fluid transport and their determinants in PD patients. *Perit. Dial. Int.* 34 195–203. 10.3747/pdi.2012.00189 24084837 PMC3968105

[B3] Combet S. Miyata T. Moulin P. Pouthier D. Goffin E. Devuyst O. (2000). Vascular proliferation and enhanced expression of endothelilal nitric oxide synthase in human peritoneum exposed to long-term peritoneal dialysis. *J. Am. Soc. Nephrol.* 11 717–728. 10752531 10.1681/ASN.V114717

[B4] Del Peso G. Jimenez-Heffernan J. A. Bajo M. A. Aroeira L. S. Aquillera L. S. Fernandez-Perpen A. (2008). Epithelial-to-mesenchymal transition of mesothelial cells is an early event during peritoneal dialysis and is associated with high peritoneal transport. *Kidney Int.* 73(Suppl. 108), S26–S33. 10.1038/sj.ki.5002598 18379544

[B5] Douma C. E. De Waart D. R. Struijk D. G. Krediet R. T. (1998). Are phospholipase A2 and nitric oxide involved in the alterations in peritoneal transport during CAPD peritonitis? *J. Lab. Clin. Med.* 132 329–340. 10.1016/S0022-2143(98)90047-6 9794705

[B6] Guyton A. C. (1981). *Textbook of Medical Physiology*, 6th Edn. Philadelphia, PA: Saunders, 358–369.

[B7] Heimburger O. Waniewski J. Werynski A. Lindholm B. (1992). A quantitative description of solute and fluid transport during peritoneal dialysis. *Kidney Int.* 41 1320–1332. 10.1038/ki.1992.196 1614047

[B8] Honda K. Nita K. Horita S. Yumura W. Nihei N. Nagai R. (1999). Accumulation of advanced glycation end products in the peritoneal vasculature of continuous ambulatory peritoneal dialysis patients with low ultrafiltration. *Nephrol. Dial. Transplant.* 14 1541–1549. 10.1093/ndt/14.6.1541 10383022

[B9] Imholz A. L. Koomen G. C. Struijk D. G. Arisz L. Krediet R. T. (1993). Effect of an increased intraperitoneal pressure on fluid and solute transport during CAPD. *Kidney Int.* 44 1078–1085. 10.1038/ki.1993.351 8264138

[B10] Imholz A. L. T. Koomen G. C. M. Struijk D. G. Arisz L. Krediet R. T. (1994). Fluid and solute transport in CAPD patients using ultralow sodium dialysate. *Kidney Int.* 46 333–340. 10.1038/ki.1994.279 7967344

[B11] Imholz A. L. T. Koomen G. C. M. Voorn W. J. Struijk D. G. Arisz L. Krediet R. T. (1978). Day-to-day variability of peritoneal fluid and solute transport in recumbent and upright position during CAPD. *Nephrol. Dial. Transplant.* 13 145–153. 9481731 10.1093/ndt/13.1.146

[B12] Kawanishi H. Kawaguchi Y. Fukui H. Hara S. Imada A. Kubo H. (2004). Encapsulating peritoneal sclerosis in Japan: a prospective, controlled, multicenter study. *Am. J. Kidney Dis.* 44 729–737. 10.1016/S0272-6386(04)00953-9 15384025

[B13] Krediet R. T. (2004). The effective lymphatic absorption rate is an accurate and useful concept in the physiology of peritoneal dialysis. *Perit. Dial. Int.* 24 309–313. 15335142

[B14] Krediet R. T. (2009). “The physiology of peritoneal solute, water and lymphatic transport,” in *Nolph and Gokal’s Textbook of Peritoneal Dialysis*, 3rd Edn, eds Khanna R. Krediet R. T. (Berlin: Springer).

[B15] Krediet R. T. Balafa O. (2010). Cardiovascular risk in the peritoneal dialysis patient. *Nat. Rev. Nephrol.* 6 451–460. 10.1038/nrneph.2010.68 20567248

[B16] Krediet R. T. Lopes Barreto D. Struijk D. G. (2016). Can free water transport be used as a clinical parameter for peritoneal fibrosis in long-term PD patients? *Perit. Dial. Int.* 36 124–128. 10.3747/pdi.2015.00129 26475849 PMC4803355

[B17] Krediet R. T. Struijk D. G. Koomen G. C. M. Arisz L. (1991). Peritoneal fluid kinetics during CAPD measured with intraperitoneal dextran 70. *ASAIO Trans.* 37 662–667. 1722690

[B18] Krediet R. T. Van Diepen A. T. N. Coester A. M. Struijk D. G. (2018). Peritoneal vasculopathy in the pathophysiology of long-term ultrafiltration failure. An hypothesis based on clinical observations. *Clin. Nephrol.* 10.5414/CN109596 [Epub ahead of print]. 30431432

[B19] Krediet R. T. Zemel D. Imholz A. L. T. Koomen G. C. M. Struijk D. G. Arisz L. (1993). Indices of peritoneal permeability and surface area. *Perit. Dial. Int.* 13(Suppl. 2), S31–S34. 8399595

[B20] Krediet R. T. Zuyderhoudt F. M. J. Boeschoten E. W. Arisz L. (1987). Alterations in the peritoneal transport of water and solutes during peritonitis in continuous ambulatory peritoneal dialysis patients. *Eur. J. Clin. Invest.* 17 43–52. 10.1111/j.1365-2362.1987.tb01224.x 3106050

[B21] La Milia V. Di Fillipo S. Crepaldi M. Dell Vecchio L. DellÓro C. Andrulli S. (2005). Mini-peritoneal equilibration test: a simple and fast metod to assess free water transport and small solute transport across the peritoneal membrane. *Kidney Int.* 68 840–846. 10.1111/j.1523-1755.2005.00465.x 16014064

[B22] Lambie M. Chess J. Donovan K. L. Kim Y. L. Do J. Y. Lee H. B. (2013). Independent effects of systemic and peritoneal inflammation on peritoneal dialysis survival. *J. Am. Soc. Nephrol.* 24 2071–2080. 10.1681/ASN.2013030314 24009237 PMC3839554

[B23] Lopes Barreto D. Sampimon D. E. Struijk D. G. Krediet R. T. (2018). Early detection of imminent encapsulating peritoneal sclerosis: free water transport, selected effluent proteins or both? *Perit. Dial. Int.* 10.3747/pdi.2017.00194 [Epub ahead of print]. 30478138

[B24] Makita Z. Radoff S. Rayfield E. J. Yang Z. Skonik E. Delaney V. (1991). Advanced glycoslation end products in patients with diabetic nephropathy. *N. Engl. J. Med.* 325 836–842. 10.1056/NEJM199109193251202 1875967

[B25] Morelle J. Snow A. Hautem N. Bouzin C. Crott R. Devuijst O. (2015). Interstitial fibrosis restricts osmotic water transport in encapsulating peritoneal sclerosis. *J. Am. Soc. Nephrol.* 26 2521–2533. 10.1681/ASN.2014090939 25636412 PMC4587704

[B26] Mujais S. Nolph K. D. Gokal R. Blake P. Burkart J. Coles G. (2000). Evaluation and management of ultrafiltration problems in peritoneal dialysis. *Perit. Dial. Int.* 20(Suppl. 4), S5–S21. 11098926

[B27] Nakayama M. Kawaguchi Y. Yamada K. Hasegawa T. Takazoe K. Katoh N. (1997). Immunohistochemical detection of advanced glycosylation end-products in the peritoneum and its possible pathophysiological role in CAPD. *Kidney Int.* 51 182–186. 10.1038/ki.1997.22 8995732

[B28] Ni J. Verbavatz J.-M. Rippe A. Boisde I. Moulin P. Rippe B. (2006). Aquaporin-1 plays an essential role in water permeability and ultrafiltration during peritoneal dialysis. *Kidney Int.* 69 1518–1525. 10.1038/sj.ki.5000285 16508653

[B29] Nolph K. D. Hano J. E. Teschan P. E. (1969). Peritoneal sodium transport during hypertonic peritoneal dialysis. *Ann. Intern. Med.* 70 931–941. 10.7326/0003-4819-70-5-931 5783428

[B30] Pannekeet M. M. Imholz A. L. T. Struijk D. G. Koomen G. C. M. Langedijk M. J. Schouten N. (1995). The stanard peritoneal permeability analysis: a tool for the assessment of peritoneal permeability characteristics in CAPD patients. *Kidney Int.* 48 866–875. 10.1038/ki.1995.363 7474677

[B31] Parikova A. Smit W. Struijk D. G. Zweers M. M. Krediet R. T. (2005). The contribution of free water transport and small pore fluid transport to the total fluid removal in peritoneal dialysis. *Kidney Int.* 68 1849–1856. 10.1111/j.1523-1755.2005.00604.x 16164663

[B32] Parikova A. Smit W. Zweers M. M. Struijk D. G. Krediet R. T. (2008). Free water transport, small pore transport and the osmotic pressure gradient. *Nephrol. Dial. Transplant.* 23 2350–2355. 10.1093/ndt/gfm768 17984106

[B33] Pecoits-Filho R. Cavalho M. J. Stenvinkel P. Lindholm B. Heimburger O. (2006). Systemic and intraperitoneal interleukin-6 system during the first year of peritoneal dialysis. *Perit. Dial. Int.* 26 53–63. 16538876

[B34] Rippe B. (1993). A three-pore model of peritoneal transport. *Perit. Dial. Int.* 12(Suppl. 2), S35–S38. 8399608

[B35] Rippe B. Stelin G. Ahlmen J. (1986). “Lymph flow from the peritoneal cavity in CAPD patients,” in *Frontiers in Peritoneal Dialysis*, eds Maher J. F. Winchester J. F. (New York, NY: Field, Rich and Associates), 224–230.

[B36] Rippe B. Stelin G. Haraldsson B. (1991). Computer simulations of peritoneal fluid transport in CAPD. *Kidney Int.* 40 315–325. 10.1038/ki.1991.216 1942781

[B37] Sampimon D. E. Coester A. M. Struijk D. G. Krediet R. T. (2011). The time course of peritoneal transport parameters in peritoneal dialysis patients who develop encapsulating peritoneal sclerosis. *Nephrol. Dial. Transplant.* 26 291–298. 10.1093/ndt/gfq343 20566569

[B38] Sampimon D. E. Lopes Barreto D. Coester A. M. Struijk D. G. Krediet R. T. (2014). The value of osmotic conductance, and free water transport in the prediction of encapsulating peritoneal sclerosis. *Adv. Perit. Dial.* 30 21–26. 25338417

[B39] Smit W. Struijk D. G. Ho-dac-Pannekeet M. M. Krediet R. T. (2004). Quantification of free water transport in peritoneal dialysis. *Kidney Int.* 66 849–854. 10.1111/j.1523-1755.2004.00815.x 15253742

[B40] Stelin G. Rippe B. (1990). A phenomenological interpretation of the variation in dialysate volume with dwell time in CAPD. *Kidney Int.* 38 465–472. 10.1038/ki.1990.227 2232489

[B41] Struijk D. G. Krediet R. T. Imholz A. L. T. Koomen G. C. M. Arisz L. (1996). Fluid kinetics in CAPD patients during dialysis with a bicarbonate-based hypoosmolar solution. *Blood Purif.* 14 217–226. 10.1159/000170264 8738535

[B42] Van Diepen A. T. N. Van Esch S. Struijk D. G. Krediet R. T. (2015). The first peritonitis episode alters the natural course of peritoneal membrane characteristics in peritoneal dialysis patients. *Perit. Dial. Int.* 35 324–332. 10.3747/pdi.2014.00277 24711641 PMC4443991

[B43] Van Esch S. Zweers M. M. Jansen M. A. M. De Waart D. R. Van Maanen J. G. Krediet R. T. (2004). Determinants of peritoneal solute transport rates in newly started non-diabetic peritoneal dialysis patients. *Perit. Dial. Int.* 24 554–561. 15559485

[B44] Williams J. D. Craig K. J. Topley N. Von Ruhland C. Fallon M. Newman G. R. (2002). Morphologic changes in the peritoneal membrane of patients with renal disease. *J. Am. Soc. Nephrol.* 13 470–479. 11805177 10.1681/ASN.V132470

[B45] Yanez-Mo M. Lara-Pezzi E. Selgas R. Ramirez-Huska M. Dominiqez-Jimenez C. Jimenez-Heffernan J. A. (2003). Peritoneal dialysis and epithelial-to-mesenchymal transition of mesothelial cells. *N. Engl. J. Med.* 348 403–413. 10.1056/NEJMoa020809 12556543

[B46] Zemel D. Koomen G. C. M. Hart A. A. M. Ten Berge R. J. M. Struijk D. G. Krediet R. T. (1993). Relationship of TNFα, interleukin-6, and prostaglandins to peritoneal permeability for macromolecules during longitudidnal follow-up of peritonitis in continuous ambulatory peritoneal dialysis. *J. Lab. Clin. Med.* 122 686–696. 8245688

[B47] Zweers M. M. De Waart D. R. Smit W. Struijk D. G. Krediet R. T. (1999). The growth factors VEGF and TGF-β1 in peritoneal dialysis. *J. Lab. Clin. Med.* 134 124–132. 10.1016/S0022-2143(99)90116-6 10444025

